# The contribution of active transportation to population physical activity levels

**DOI:** 10.24095/hpcdp.45.5.03

**Published:** 2025-05

**Authors:** Stephanie A. Prince, Gregory P. Butler

**Affiliations:** 1 Centre for Surveillance and Applied Research, Public Health Agency of Canada, Ottawa, Ontario, Canada; 2 School of Epidemiology and Public Health, Faculty of Medicine, University of Ottawa, Ottawa, Ontario, Canada

**Keywords:** exercise, transportation, public health surveillance

## Abstract

We explored the contribution of active (nonmotorized) transportation, including walking and cycling, to physical activity (PA) levels and its association with PA recommendations adherence (youth: ≥60 min/day; adults: ≥150 min/week) using self-reported domain-specific and accelerometer-measured PA from Cycles 4 to 6 (2014–2019) of the Canadian Health Measures Survey (N=8620). Recreation and household or occupational PA were similar for users and non-users, but accelerometer-measured PA was significantly higher among active transportation users (12–17 years: 56.6 vs. 47.7 min/day; 18–64 years: 33.4 vs. 22.8 min/day, 65–79 years: 21.5 vs. 13.7 min/day). Active transportation was not associated with meeting the PA recommendation for youth after adjusting for confounders (adjusted odds ratio [aOR] = 1.39; 95% confidence interval [CI]: 0.91–2.11), but it was for adults (18–64 years: aOR = 2.71, 95% CI: 2.18–3.37; 65–79 years: aOR = 2.26, 95% CI: 1.39–3.69). Given its contribution to population PA levels, supporting active transportation should be considered an important tool for health promotion.

**Highlights**

- Recreation and household or occupational physical activity were similar for active transportation users and non-users.
- Accelerometer-measured physical activity was higher among active transportation users across all age groups.
- Participating in active transportation increases the likelihood of achieving the physical activity recommendation for adults and is important for health promotion.

## Introduction

Physical activity (PA) is protective against many chronic conditions and all-cause mortality and promotes positive mental health and well-being.^1^-^3^ Measuring PA across several domains, including recreational, occupational or school, household and transportation domains, is important for population surveillance.^4^,^5^ Although PA promotion often prioritizes leisure PA, largely because most of the earlier evidence showing the benefits of PA for health based on studies with self-reported leisure PA,^6^-^8^ examination of all domains provides critical information to informing PA promotion activities.

Active transportation, or self-powered or nonmotorized travel, involves walking, cycling or other means of getting to destinations like work or school, running errands or shopping for groceries, visiting friends, going to places of entertainment and various other trips.^9^ Data from the 2021 Canadian Community Health Survey show that 61.0% of youth and 41.7% of adults engage in some form of active transportation to get to destinations.^5^ The 2021 Canadian Census (collected during the COVID-19 pandemic) recorded that 6.2% of working Canadians reported using active transportation as their main mode of commuting to work (down from 6.9% in 2016).^10^ An additional 1.7% and 1.3% reported walking and cycling, respectively, when they used multiple modes of transportation to commute.^10^

Active transportation is a valuable and often overlooked tool for achieving the PA recommendations identified in Canada’s 24-Hour Movement Guidelines.^11^ Specifically, the guidelines recommend that adults achieve 150 minutes of weekly moderate-to-vigorous intensity physical activity (MVPA) and children and youth engage in an average of 60 minutes of MVPA per day. Active transportation is associated with higher levels of PA and an increased likelihood of achieving PA recommendations.^12^ Further, it is likely that active transportation promotes additional PA rather than replacing other forms of PA.^12^ Previous studies conducted in Canada (largely using nonrepresentative samples) also suggested that active transportation, including walking to and from public transit, results in a greater likelihood of meeting the PA recommendations,^13^-^15^ but with some evidence of compensation among older adults.^16^ Transportation-related PA (especially cycling) is associated with better cardiometabolic health^17^ and reduced risk of cardiovascular disease,^18^-^21^ type 2 diabetes,^21^,^22^ cancer-related mortality^18^ and all-cause mortality.^18^-^21^

Most of the research exploring the association between active transportation and PA has emerged from Australia, Europe and the United States.^12^ The objective of this study was to determine the association between active transportation and PA recommendations and to understand the contribution of active transportation to Canadians’ overall PA levels.

## Methods

This study combined data from Cycles 4 to 6 (2014–2019) of the Canadian Health Measures Survey (CHMS). The CHMS is an ongoing cross-sectional survey, conducted by Statistics Canada, that collects self-reported and directly measured health information from a nationally representative sample of the household-dwelling population aged 3 to 79 years in Canada.^23^ Excluded are people living in the three territories and certain remote regions, on reserves and other Indigenous settlements, and in institutions, which equates to approximately 4% of the target population.^23^ The CHMS is collected year-round across all seasons.

The analyzed sample included 8620 youth and adults (aged 12–79 years) with complete and valid self-reported and accelerometer-measured PA.

Youth respondents were asked about the number of minutes per day they spent doing different types of MVPA over the previous 7 days. Adult respondents were asked about the number of minutes they spent doing different types of MVPA in the previous 7 days for a minimum of 10 continuous minutes. Self-reported average minutes per day spent in total and domain-specific MVPA was estimated for transportation, recreational and occupational or household PA. “Active transportation users” were defined as those people who used nonmotorized ways, such as walking or cycling, to get to school, work, the bus stop, and so on, or for running errands, going shopping or visiting friends.

After completing the household section of the CHMS questionnaire, respondents who attended a clinic visit were asked to wear an Actical accelerometer (Philips Respironics, Oregon, US) over their right hip during waking hours for 7 consecutive days. A minimum of 4 days with 10 hours or more of wear time per day was required. Previously validated movement intensity thresholds^24^,^25^ were applied to derive time spent being sedentary and being physically active at light, moderate and vigorous intensity PA. PA recommendation adherence was defined as an average of 60 or more minutes per day and 150 or more minutes per week of accelerometer-measured MVPA for youth and adults, respectively.^11^

Adherence to the PA recommendation was estimated using proportions and 95% confidence intervals (CIs) and compared between active transportation users and non-users using the Rao-Scott chi-square (χ^2^)test. We used multivariate logistic regression to determine the association between self-reported active transportation use and PA recommendation adherence for youth (12–17 years) and adults (18–64 and 65–79 years) while adjusting for age, sex, income quintile and ethnicity (not racialized, racialized, Indigenous Peoples).

*T* tests compared time spent in self-reported PA domains and accelerometer-measured MVPA among active transportation users and non-users. All measures of PA were log-transformed after adding a constant to address the high number of zeroes. All analyses were weighted using combined cycle accelerometer subsample survey weights. Degrees of freedom were set at 33. To account for survey design effects, 95% CIs were estimated using the bootstrap-balanced repeated replication technique with 500 replicate weights. Statistical significance was set at *p*<0.05. Statistical analyses were performed using SAS Enterprise Guide version 7.1 (SAS Institute Inc., Cary, NC, US).

The CHMS protocol was approved by the Health Canada–Public Health Agency of Canada Research Ethics Board.

## Results

**Figure 1:**
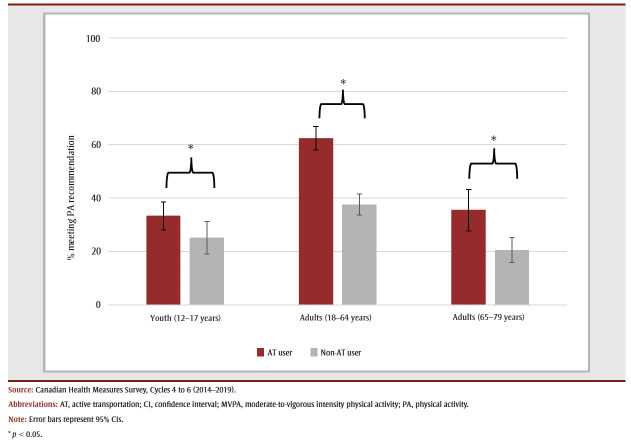
Adherence to the PA recommendation (≥ 150 min/week of accelerometer-measured MVPA) by active transportation–using and non-using youth (12–17 years) and adults (18–64 and 65–79 years), Canada (excluding territories)

Almost three-quarters (70.5%) of youth, less than half (44.2%) of adults aged 18 to 64 years and one-third (33.8%) of adults aged 65 to 79 years used active means of transportation. Across all ages, adherence to the PA recommendation was significantly higher among active transportation users than non-users (12–17 years: 33.3 vs. 25.1%; 18–64 years: 62.5 vs. 37.6%; 65–79 years: 35.5 vs. 20.5%) (see Figure 1).

While the PA recommendation adherence point estimate was higher among active transportation–using youth, active transportation was not associated with meeting the PA recommendation after adjusting for confounders (adjusted odds ratio [aOR] = 1.39, 95% confidence interval [CI]: 0.91–2.11). Adult active transportation users were significantly more likely than adult non-users to meet the PA recommendation, even after adjusting for confounders (18–64 years: aOR = 2.71, 95% CI: 2.18–3.37; 65–79 years: aOR=2.26, 95% CI: 1.39–3.69). Further adjustment for season of response had little effect on the effect estimates, and season was not a significant predictor in the models (results not shown).

**Figure 2:**
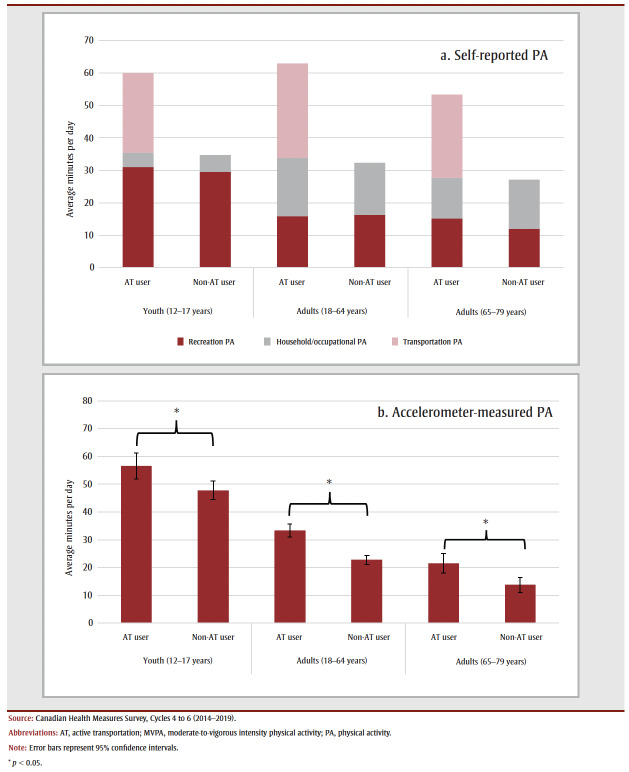
Average minutes per day spent in (a) different self-reported PA domains and (b) accelerometer-measured MVPA by active transportation–using and non-using youth (12–17 years) and adults (18–64 and 65–79 years), Canada (excluding territories)

Average time spent in recreation and occupational or household PA did not differ statistically between active transportation users and non-users, while time spent in accelerometer-measured MVPA was significantly higher for active transportation users than non-users across all age groups (12–17 years: 56.6 vs. 47.7 min/day; 18–64 years: 33.4 vs. 22.8 min/day; 65–79 years: 21.5 vs 13.7 min/day) (see Figure 2).

## Discussion

The results of this study suggest that participating in active transportation increases the likelihood of achieving the PA recommendations of the 24-Hour Movement Guidelines among adults. Canadians that engaged in active transportation had similar levels of occupational or household and recreational PA, but higher levels of accelerometer-measured MVPA. As such, active transportation appears to be additive rather than substitutive across PA domains.

Recent estimates show that only 49.2% of adults and 35.6% of youth in Canada, adhere to the PA recommendations.^5^ Walking is generally more prevalent than cycling, males report more time spent in active transportation than do females, and active transportation use declines with age.^9^ Our findings are similar to recent systematic reviews that suggested that those who engage in active transportation also engage in more device-measured total PA and that active transportation does not generally displace other activity.^12^,^26^,^27^

The World Health Organization’s Global Action Plan on Physical Activity 2018–2030 has a target reduction of 15% in the global prevalence of physical inactivity among adolescents and adults by 2030.^28^ The plan calls for investments in policies and urban and transport planning to promote walking and cycling and other forms of active transportation.^28^ *A Common Vision for Increasing Physical Activity and Reducing Sedentary Living in Canada: Let’s Get Moving*^29^ and the 2017 Chief Public Health Officer of Canada’s Report^30^ recognized the importance of the physical environment for providing supportive and accessible opportunities to integrate PA into the daily lives of Canadians. One of the strategic imperatives of the *Common Vision* identifies supporting active transportation and transit solutions (e.g. enhancing bike routes, integrating public transportation systems).^29^ Canadian research has shown that the active living friendliness or walkability of a neighbourhood is positively associated with accelerometer-measured MVPA and active transportation–based PA by youth and adults.^31^ Systematic review evidence has also shown that the creation and improvement of infrastructure that supports pedestrians and cyclists (e.g. sidewalks, street connectivity, bike paths, traffic calming zones) is positively associated with active transportation.^32^-^34^

The literature showing causal effects of supportive infrastructure on active transportation suggests that investments in sidewalks and cycling infrastructure, for example, by the transportation or infrastructure sectors, could also be viewed as public health investments.^32^ The National Active Transportation Strategy^35^ outlines a vision for advancing active transportation through evidence-based investments in new and existing infrastructure that supports active transportation. 

The perceived comfort and safety of infrastructure is a key consideration for promoting active transportation.^36^,^37^ The Canadian Bikeway Comfort and Safety (Can-BICS) classification system provides a standard naming convention for cycling infrastructure across cities in Canada and a three-tier classification system based on the safety and comfort of people using bicycle facilities.^38^ Research using 2022 OpenStreetMap data found that 34% of Canadian neighbourhoods had no cycling infrastructure, 40% had no medium- or high-comfort cycling infrastructure and only 5% (for 6% of the population) had the highest category of Can-BICS.^39^ Improving the safety and comfort of walking and cycling infrastructure in Canada is likely an important way of promoting active transportation and contributing to the daily PA of Canadians and hence their health.

***Strengths and limitations***

This study includes data from a nationally representative sample of Canadians with measured and self-reported PA. In our assessment of the association between active transportation and PA recommendation adherence, we adjusted for important sociodemographics that have shown to be associated with PA and active transportation (i.e. age, sex, income quintile and ethnicity). In addition, a sensitivity analysis assessed whether season of data collection affected the estimates. It is possible, however, that the results could be affected by residual confounding from variables not included in the models.

Important limitations of this work need to be acknowledged. There is a discordance between the self-reported and accelerometer-measured PA data; the self-reported PA data reflect a 7-day recall in the week before the household questionnaire, while the accelerometer data are collected the week after the visit to the clinic. It is also possible that those who wore an accelerometer changed their PA habits as a result of being monitored. In addition, comparisons of self-reported and accelerometser-measured total PA levels (Figure 2) indicate that participants who engaged in active transportation may overreport their PA levels. Previous work has also found that self-reported and accelerometer-measured levels of PA differ.^40^,^41^ Future work could explore whether engaging in certain types of PA biases this discordance.

Finally, the survey questions group walking, cycling and other forms of active transportation together. It would be important for future work, if possible, to explore the separate contributions of walking, cycling and other modes of active transportation to population PA levels. 

## Conclusion

Active transportation is an important domain for health promotion and provides important health benefits. Canadians, and especially adults, who engaged in active forms of transportation were more likely to adhere to the PA recommendations and engaged in more PA than those who do not. Policy and programs developed to promote PA should include active transportation as a core component.

## Acknowledgements

The authors would like to thank Robert Geneau, Marisol T. Betancourt and Karen C. Roberts for their review of the manuscript.

## Funding

None.

## Conflicts of interest

The authors have no conflicts of interest to disclose. Dr. Robert Geneau, Editor-in-Chief, was not involved in the deliberation of this article as he provided a critical review of the manuscript.

## Authors’ contributions and statement

SAP: Conceptualization, formal analysis, methodology, validation, writing – original draft, writing – review and editing.

GPB: Conceptualization, methodology, validation, writing – original draft, writing – review and editing.

Both authors read and approved the final version of the manuscript.

The content and views expressed in this article are those of the authors and do not necessarily reflect those of the Government of Canada.
